# Roles of nonmyogenic mesenchymal progenitors in pathogenesis and regeneration of skeletal muscle

**DOI:** 10.3389/fphys.2014.00068

**Published:** 2014-02-24

**Authors:** Akiyoshi Uezumi, Madoka Ikemoto-Uezumi, Kunihiro Tsuchida

**Affiliations:** ^1^Division for Therapies against Intractable Diseases, Institute for Comprehensive Medical Science, Fujita Health UniversityAichi, Japan; ^2^Department of Regenerative Medicine, National Center for Geriatrics and Gerontology, National Institute for Longevity SciencesAichi, Japan

**Keywords:** mesenchymal progenitors, satellite cells, PDGFRα, adipogenesis, fibrosis, heterotopic ossification, muscle regeneration, muscle atrophy

## Abstract

Adult skeletal muscle possesses a remarkable regenerative ability that is dependent on satellite cells. However, skeletal muscle is replaced by fatty and fibrous connective tissue in several pathological conditions. Fatty and fibrous connective tissue becomes a major cause of muscle weakness and leads to further impairment of muscle function. Because the occurrence of fatty and fibrous connective tissue is usually associated with severe destruction of muscle, the idea that dysregulation of the fate switch in satellite cells may underlie this pathological change has emerged. However, recent studies identified nonmyogenic mesenchymal progenitors in skeletal muscle and revealed that fatty and fibrous connective tissue originates from these progenitors. Later, these progenitors were also demonstrated to be the major contributor to heterotopic ossification in skeletal muscle. Because nonmyogenic mesenchymal progenitors represent a distinct cell population from satellite cells, targeting these progenitors could be an ideal therapeutic strategy that specifically prevents pathological changes of skeletal muscle, while preserving satellite cell-dependent regeneration. In addition to their roles in pathogenesis of skeletal muscle, nonmyogenic mesenchymal progenitors may play a vital role in muscle regeneration by regulating satellite cell behavior. Conversely, muscle cells appear to regulate behavior of nonmyogenic mesenchymal progenitors. Thus, these cells regulate each other reciprocally and a proper balance between them is a key determinant of muscle integrity. Furthermore, nonmyogenic mesenchymal progenitors have been shown to maintain muscle mass in a steady homeostatic condition. Understanding the nature of nonmyogenic mesenchymal progenitors will provide valuable insight into the pathophysiology of skeletal muscle. In this review, we focus on nonmyogenic mesenchymal progenitors and discuss their roles in muscle pathogenesis, regeneration, and homeostasis.

## Introduction

The most fundamental roles of skeletal muscle are the generation of force and the control of body movement by contraction. In addition, skeletal muscle serves as physical safeguard for other organs as it is anatomically located immediately beneath the skin. Because of its functional roles, skeletal muscle represents one of the most frequently damaged organs in the body. Therefore, regeneration from the damage is an essential property of skeletal muscle. One of the best examples of remarkable regeneration capacity of skeletal muscle is the study that showed myofiber regeneration after more than 20 repeated injuries (Sadeh et al., 1985). The high regeneration capacity of skeletal muscle is attributed to satellite cells, which reside between the sarcolemma and the basal lamina of myofibers. M-cadherin and Pax7 are the most reliable markers for mouse satellite cells (Irintchev et al., 1994; Seale et al., 2000). An essential role for satellite cells in adult myogenesis was exquisitely demonstrated by studies using genetically-engineered mice in which Pax7-expressing satellite cells are ablated (Lepper et al., 2011; Murphy et al., 2011; Sambasivan et al., 2011). These mice showed complete loss of regenerative response and severe fatty and fibrous degeneration after muscle injury, indicating that nonsatellite cells cannot compensate for satellite cell-dependent myogenesis. In addition to contributing to new myofiber formation, a subset of satellite cells have been shown to be capable of self-renewal (Collins et al., 2005; Montarras et al., 2005; Sacco et al., 2008; Rocheteau et al., 2012). Thus, satellite cells act as adult muscle stem cells.

Despite the presence of well-established stem cells that underlie the exceptional regeneration potential, skeletal muscle is replaced by ectopic tissues such as adipose tissue, fibrous connective tissue, and bone in several pathological conditions. These ectopic tissues become an aggravating factor in muscle weakness because they lack contractile ability and hinder the supply of nutrients to myofibers. In most cases, ectopic tissue formation within skeletal muscle is associated with severe destruction of myofibers, leading to the concept that dysregulation of the fate switch in satellite cells may underlie these degeneration processes. However, this concept was refuted by recent studies describing the identification of nonmyogenic mesenchymal progenitors in skeletal muscle. These studies established the pathological relevance of nonmyogenic mesenchymal progenitors to muscle diseases. In addition to their roles in disease conditions, nonmyogenic mesenchymal progenitors appear to play roles in muscle regeneration and in steady-state muscle homeostasis. This review focuses on nonmyogenic mesenchymal progenitors and describes their roles in the pathogenesis, regeneration, and homeostasis of skeletal muscle.

## Fat infiltration, fibrosis, and heterotopic ossification in skeletal muscle

In normal healthy muscle, especially in the gastrocnemius, occasional adipocytes can be encountered in connective tissue septa (Carpenter and Karpati, 2001). Physiological significance of these occasional adipocytes in normal muscle is largely unknown. Disease in which there is loss of muscle cells without efficient regeneration leads to an increase of ectopic adipocytes within fascicles (Carpenter and Karpati, 2001). In myopathic disorders accompanied by myofiber destruction, endomysial fibrous connective tissue increases from the onset of the disease, but the increase in endomysial fatty connective tissue is observed only after there has been an extensive loss of myofibers (Banker and Engel, 2004). The most striking accumulation of adipocytes is seen in advanced cases of Duchenne muscular dystrophy (DMD) or myotonic dystrophy, where a muscle may be almost entirely replaced by adipose tissue (Carpenter and Karpati, 2001; Banker and Engel, 2004). Fat infiltration in skeletal muscle is also pronounced in other disease conditions including obesity (Goodpaster et al., 2000; Greco et al., 2002; Sinha et al., 2002), type II diabetes (Goodpaster et al., 2000; Hilton et al., 2008), unloading (Manini et al., 2007), hemiparetic stroke (Ryan et al., 2002), and spinal cord injury (Gorgey and Dudley, 2007). Aging is accompanied by deterioration in muscle function, and fat infiltration in skeletal muscle increases with age Visser et al., 2002, 2005; Kim et al., 2009; Marcus et al., 2010; Kragstrup et al., 2011. It is not known whether adipocytes within skeletal muscle would disappear if the disease conditions could be reversed.

Fibrosis is a pathological feature associated with many chronic inflammatory diseases. Fibrosis is defined by the excessive accumulation of extracellular matrix (ECM) components, which can lead to permanent scarring and organ malfunction. Although ECM deposition is an indispensable and, typically, reversible part of wound healing, the repair process can produce a progressively irreversible fibrosis if the tissue injury is severe or repetitive or if there is a defect in the repair machinery. Fibrosis in skeletal muscle can be seen in most conditions where there is chronic muscle damage or insufficient regeneration but is most prominent in muscular dystrophy (Carpenter and Karpati, 2001; Banker and Engel, 2004). A possible exception is myotonic dystrophy, where fat infiltration appears to proceed without much fibrosis (Carpenter and Karpati, 2001). Sports injuries such as lacerations, contusions or strains can also elicit irreversible fibrotic response and lead to scar formation depending on the severity of injuries (Jarvinen et al., 2000; Beiner and Jokl, 2001). Although myofibroblasts are cells responsible for fibrosis of other organs such as liver and kidney, one should keep in mind that these cells have not been identified in most skeletal muscle diseases except for nodular fasciitis (Wirman, 1976) and pseudomalignant myositis ossificans (Povysil and Matejovsky, 1979).

Heterotopic ossification (HO) is defined as the abnormal formation of mature, lamellar bone in soft tissues outside the skeletal periosteum. The most commonly affected site is skeletal muscle. HO has been thought to result from inappropriate differentiation of progenitor cells that is induced by a pathological imbalance of local or systemic factors. There are three recognized etiologies of HO: traumatic, neurogenic, and genetic (Balboni et al., 2006). Traumatic HO is the most common type of HO and typically recognized after fractures, severe burns, and surgical trauma, especially after total hip arthroplasty (Nilsson and Persson, 1999). Neurogenic HO can be frequently seen after injuries to central nervous system (Garland et al., 1980). Other neurologic conditions have also been implicated in the development of HO, including meningitis (Lorber, 1953), myelitis (Stoikovic et al., 1955), and tetanus (Ishikawa et al., 1982). Genetic disorders in which HO arises in skeletal muscle are fibrodysplasia ossificans progressiva (FOP) and progressive osseous heteroplasia (POH) (Kaplan and Shore, 2000; Pignolo et al., 2011). FOP is a severely disabling heritable disorder of connective tissue characterized by congenital malformations of the great toes and progressive extraskeletal ossification. Mutation of the *ALK-2* gene, a BMP type I receptor, was identified in FOP patients (Shore et al., 2006) and has been shown to contribute to the pathogenesis of FOP (Chakkalakal et al., 2012). POH is a genetic disorder of mesenchymal differentiation characterized by dermal ossification during infancy and progressive HO of cutaneous, subcutaneous, and deep connective tissues including skeletal muscle during childhood. An inactivating mutation of *GNAS1* gene was reported to be the cause of POH (Shore et al., 2002).

## Identification of nonmyogenic mesenchymal progenitors and their contribution to pathogenesis of skeletal muscle

**Figure 1:**
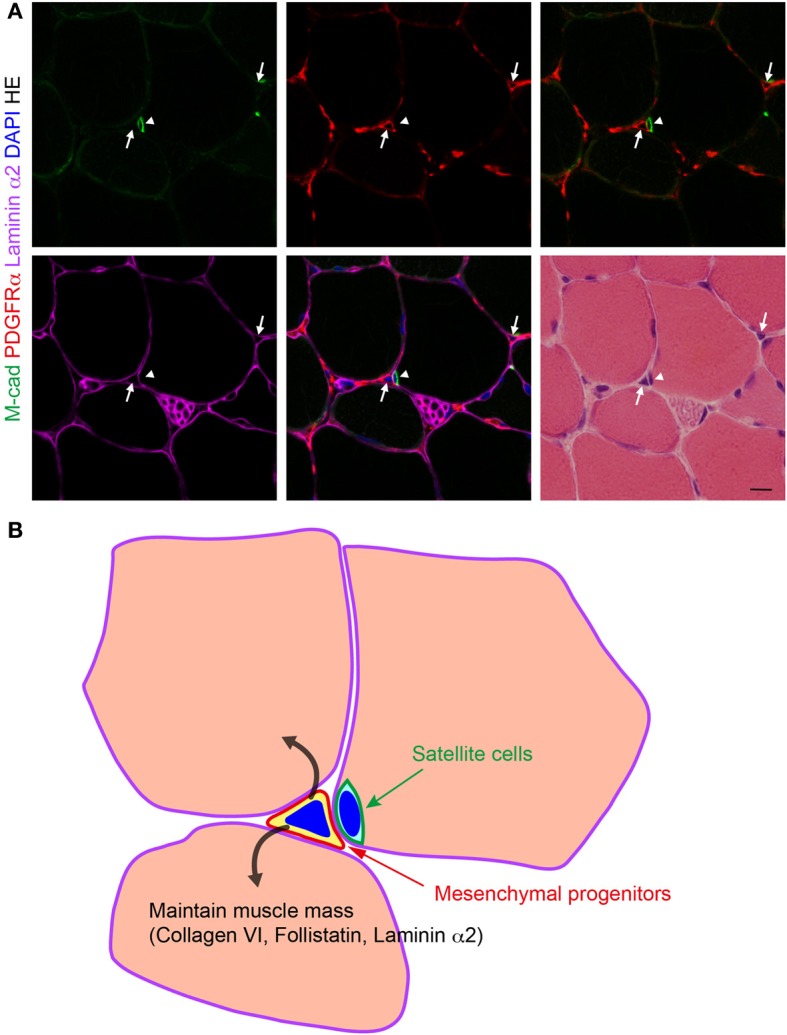
**Localization of PDGFRα^+^ mesenchymal progenitors in normal muscle. (A)** Fresh frozen section of mouse TA muscle subjected to immunofluorescence staining for M-cadherin (M-cad), PDGFRα, and laminin α2, and subsequently to HE staining. Arrows indicate PDGFRα^+^ mesenchymal progenitors and arrowhead indicates satellite cells. Scale bar: 10 μm. **(B)** Schematic view of **(A)**. Mesenchymal progenitors that reside in muscle interstitium express collagen VI, follistatin, and laminin α2, and maintain muscle mass in a normal physiological condition.

To clarify the origin of cell populations involved in the fatty degeneration of skeletal muscle, we conducted a comprehensive survey of cells that reside in skeletal muscle using a FACS-based cell isolation technique. As a consequence, we found that only cells expressing PDGFRα can differentiate into adipocytes. In addition to adipogenic potential, PDGFRα^+^ cells can differentiate into osteoblastic or smooth muscle-like cells but scarcely differentiate into skeletal muscle lineage cells. Therefore, we termed these cells mesenchymal progenitors (Uezumi et al., 2010). Mesenchymal progenitors reside in the muscle interstitium and therefore represent a cell population that is distinct from satellite cells (Figure 1). These cells are more frequently observed in the perimysium than in the endomysium, particularly in the perivascular space. But they are distinct from pericytes because they reside outside the vessel wall and outside the capillary basement membrane. They do not originate from bone marrow, but instead represent a cell population that is resident in skeletal muscle. Importantly, only mesenchymal progenitors can participate in ectopic fat cell formation when transplanted into fatty degenerating muscle, while other cells residing within skeletal muscle do not have such an activity (Uezumi et al., 2010). Another group also identified cells with adipogenic potential on the basis of Sca-1 and CD34 expression (Joe et al., 2010). Sca-1^+^CD34^+^ cells were referred to as fibro/adipogenic progenitors (FAPs), because these cells have the potential to produce both adipocytes and fibroblasts but fail to differentiate into osteogenic cells *in vitro*. PDGFRα^+^ mesenchymal progenitors express Sca-1, and complementally FAPs express PDGFRα (Joe et al., 2010; Uezumi et al., 2010); thus both cell types seem to represent the same cell population. Adipocytes that emerge in skeletal muscle show unilocular morphology and PDGFRα^+^ cell-derived adipocytes express high level of *Leptin* (Uezumi et al., 2010), indicating that mesenchymal progenitors have a high propensity to differentiate into white adipose lineage. However, brown adipogenic potential of Sca-1^+^ progenitors was also demonstrated (Schulz et al., 2011). Thus, mesenchymal progenitors appear to possess the capacity to differentiate into both white and brown adipocytes.

**Figure 2:**
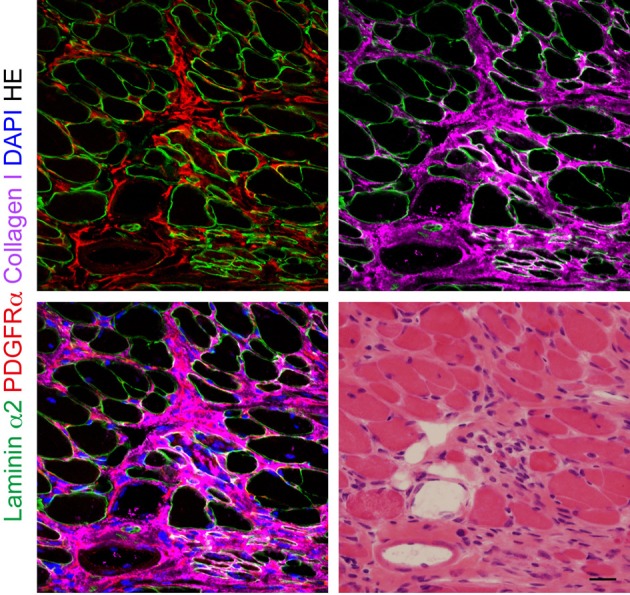
**The behavior of PDGFRα^+^ mesenchymal progenitors in dystrophic muscle.** Fresh frozen section of mdx diaphragm subjected to immunofluorescence staining for laminin α2, PDGFRα, and collagen I, and subsequently to HE staining. Scale bar: 20 μm.

In the subsequent study, we revealed that mesenchymal progenitors also contribute to skeletal muscle fibrosis (Uezumi et al., 2011). A striking increase in the number of PDGFRα^+^ cells is conspicuous in fibrotic areas of the diaphragm from mdx mice (Figure 2). Using an irradiation-induced muscle fibrosis model, we further demonstrated that transplanted PDGFRα^+^ cells directly participate in fibrotic scar tissue formation with negligible myogenic activity (Uezumi et al., 2011). In contrast, satellite cell-derived myoblasts exclusively participate in myofiber formation but do not contribute to fibrous connective tissue formation. A study by Dulauroy et al. provided further details. By inducible lineage tracing, Dulauroy et al. showed that a subset of PDGFRα^+^ cells begin to express ADAM12 during muscle injury and an ADAM12^+^PDGFRα^+^ subset accumulates fibrotic regions of injured muscle (Dulauroy et al., 2012). This study sophisticatedly demonstrated that endogenous mesenchymal progenitors are indeed the origin of profibrotic cells. However, the study utilized a CTX muscle injury model. Because skeletal muscle regenerates almost completely without development of fibrotic scar tissue after CTX injection (Hawke and Garry, 2001; Harris, 2003), this model reflects a reversible repair process rather than irreversible fibrosis. Therefore, the behavior of ADAM12^+^PDGFRα^+^ cells in a more pathologically relevant model would be of considerable interest. Using single myofibers isolated from Pax7-CreER/ROSA26 mice, a strain in which tamoxifen administration leads to permanent β-galactosidase expression only in Pax7^+^ satellite cells, satellite cells were shown to become fibrogenic under the influence of aged serum (Brack et al., 2007). The conversion ratio was around 10% in this context. In contrast, nearly 100% of PDGFRα^+^ mesenchymal progenitors were converted to fibrogenic cells when treated with TGF-β at a concentration that had no effect on satellite cell myogeneity (Uezumi et al., 2011). Although different experimental conditions used in those studies make direct comparisons difficult, mesenchymal progenitors seem to be more prone to fibrogenic conversion than satellite cells. However, further studies are needed to elucidate which cell type—satellite cells or PDGFRα^+^ mesenchymal progenitors—is the main source of fibrogenic cells in an aged environment *in vivo*. The link between fibrogenesis and PDGFRα signaling has been demonstrated by several studies. Olson and Soriano generated mice in which mutant PDGFRα with increased kinase activity was knocked into a PDGFRα locus (Olson and Soriano, 2009). Thus, constitutively active PDGFRα signaling is operative only in cells that express PDGFRα endogenously in the mutant mice. The mice with mutant PDGFRα showed progressive fibrosis in multiple organs including skeletal muscle. We showed that stimulation of PDGFRα signaling in PDGFRα^+^ mesenchymal progenitors promotes cell proliferation and upregulates the expression of fibrosis-related genes (Uezumi et al., 2011). Imatinib, an inhibitor of several tyrosine kinases including PDGFR, c-kit, and bcr-abl oncogene, was shown to ameliorate the dystrophic symptoms of mdx mice (Bizario et al., 2009; Huang et al., 2009; Ito et al., 2013). Importantly, imatinib treatment decreased the phosphorylation level of PDGFRα in the dystrophic muscle (Huang et al., 2009), and treatment of PDGFRα^+^ mesenchymal progenitors with imatinib inhibited PDGFRα-induced proliferation and expression of fibrotic genes (Ito et al., 2013). Taking these findings into account, the therapeutic effect of imatinib exerted on dystrophic mice seems to be achieved at least in part through targeting PDGFRα^+^ mesenchymal progenitors.

The contribution of mesenchymal progenitors to HO in skeletal muscle was exquisitely demonstrated by Goldhamer and colleagues. By crossing lineage-specific Cre mice with Cre-dependent reporter mice, they generated several mouse lines in which specific lineage cells were permanently labeled, and showed that skeletal myogenic cells including satellite cells or smooth muscle cells do not contribute to BMP-induced HO. Instead, they found a significant contribution of Tie2-lineage cells to HO in two mouse models of dysregulated BMP signaling (Lounev et al., 2009). Tie2 is usually used as a marker of endothelial cells, but expression of the *Tie2* gene is also recognized in mesenchymal progenitors (Uezumi et al., 2010). To clarify which cell type labeled by Tie2-Cre contributes to the development of HO, Wosczyna et al. conducted further detailed research. Although endothelial cells constitute a large part of the Tie2^+^ fraction, they did not detectably contribute to HO. After careful cell fractionation by FACS, PDGFRα^+^Sca-1^+^ cells within Tie2^+^ fraction were found to be the predominant source of progenitors that give rise to ectopic cartilage and bone (Wosczyna et al., 2012). Clonal analysis revealed that Tie2^+^PDGFRα^+^Sca-1^+^ progenitors are multipotent as colonies derived from single Tie2^+^PDGFRα^+^Sca-1^+^ cells exhibited both osteogenic and adipogenic differentiation potentials.

As described above, several studies independently reported nonmyogenic mesenchymal progenitors that contribute to the pathogenesis of skeletal muscle. Progenitor cells described by different groups share a common cell surface phenotype: CD31^−^CD45^−^Sca-1^+^PDGFRα^+^, and therefore seem to be closely related to each other or represent the same cell population. FAPs were so named because they did not differentiate into osteogenic cells (Joe et al., 2010). But it is obvious that these progenitors possess osteo/chondrogenic potential as demonstrated in the studies by Goldhamer and colleagues. Because osteogenic potential of FAPs was assessed only in *in vitro* condition without BMP (Joe et al., 2010), their differentiation potential was probably underestimated. Therefore, the name FAPs does not represent the nature of these progenitors adequately. Although Sca-1 is widely used to identify progenitor populations in mice, it is also highly expressed in nonprogenitor cells such as endothelial cells. Furthermore, there is no human homolog of Sca-1 (Holmes and Stanford, 2007). Thus, this marker lacks relevance to the pathophysiology of human skeletal muscle. In contrast, PDGFRα is highly specific to mesenchymal progenitors (Uezumi et al., 2010) and is conserved in humans. In fact, cells equivalent to mouse PDGFRα^+^ mesenchymal progenitors can be isolated from human muscle using PDGFRα as the marker (Oishi et al., 2013). As we have consistently used this marker since we described PDGFRα as the specific marker of mesenchymal progenitors for the first time, we believe that PDGFRα is the best marker to identify mesenchymal progenitors in skeletal muscle.

## Satellite cells: muscle stem cells or multipotent stem cells?

Although satellite cells have been considered as monopotential precursors that give rise only to cells of myogenic lineage (Bischof, 2004), several studies have shown that satellite cells can differentiate into cells of nonmyogenic lineages using satellite cell-derived myoblast culture or single myofiber culture (Asakura et al., 2001; Wada et al., 2002; Shefer et al., 2004). In myoblast culture, cells are usually purified by a preplating method or by culturing muscle-derived cells at a density that selectively promotes myogenic colony formation while nonmyogenic cells grow poorly. These methods require relatively long culture periods to obtain a pure culture. However, long-term culture or clonal expansion can elicit spontaneous transformations that lead to generation of the differentiation-defective cells often observed in myogenic cell lines (Lim and Hauschka, 1984). Moreover, only a few passages significantly reduce the muscle reconstitution ability of satellite cells (Montarras et al., 2005; Ikemoto et al., 2007). Therefore, the cells obtained may have undergone considerable changes during long culture periods, and thus cannot be considered equivalent to satellite cells. Single myofiber culture is a method to isolate single myofibers with their associated satellite cells by appropriate enzymatic treatment. Because each single myofiber carries a small number of satellite cells (approximately 10–20 satellite cells depending on the muscle from which the myofiber is derived) (Collins et al., 2005), contamination with only a few nonsatellite cells will have a considerable impact. In fact, it has been demonstrated that all the adipocytes that emerge in a single myofiber culture are derived from contaminated nonsatellite cells (Starkey et al., 2011).

The studies describing nonmyogenic mesenchymal progenitors showed that satellite cells do not adopt nonmyogenic fates but exclusively contribute to myogenesis even when transplanted into degenerating muscle that facilitate adipogenic, fibrogenic, or osteo/chondrogenic differentiation (Uezumi et al., 2010, 2011; Wosczyna et al., 2012). These findings suggest that satellite cells are committed to the myogenic lineage. This was further supported by the studies showing the expression of myogenic determination genes in satellite cells or progenitors of satellite cells. Using lineage tracing or selective cell ablation strategies, it has been shown that essentially all adult satellite cells originate from progenitors that had expressed MyoD, a key muscle determination gene, prenatally, and these MyoD-expressing progenitors are essential for skeletal myogenesis and satellite cell development (Kanisicak et al., 2009; Wood et al., 2013). Myf5, another muscle determination gene, is expressed at the mRNA level in the majority of satellite cells, but its translation was shown to be repressed by miR-31, leading to a model in which posttranscriptional mechanisms hold quiescent satellite cells poised to enter the myogenic program (Crist et al., 2012). Taken together, satellite cells should be considered as muscle stem cells committed to myogenic lineage. Although recent study showed that satellite cells can differentiate into brown adipocytes, this differentiation pathway is inhibited by miR-133 in a physiological context (Yin et al., 2013).

## Roles for nonmyogenic mesenchymal progenitors in muscle regeneration

**Figure 3:**
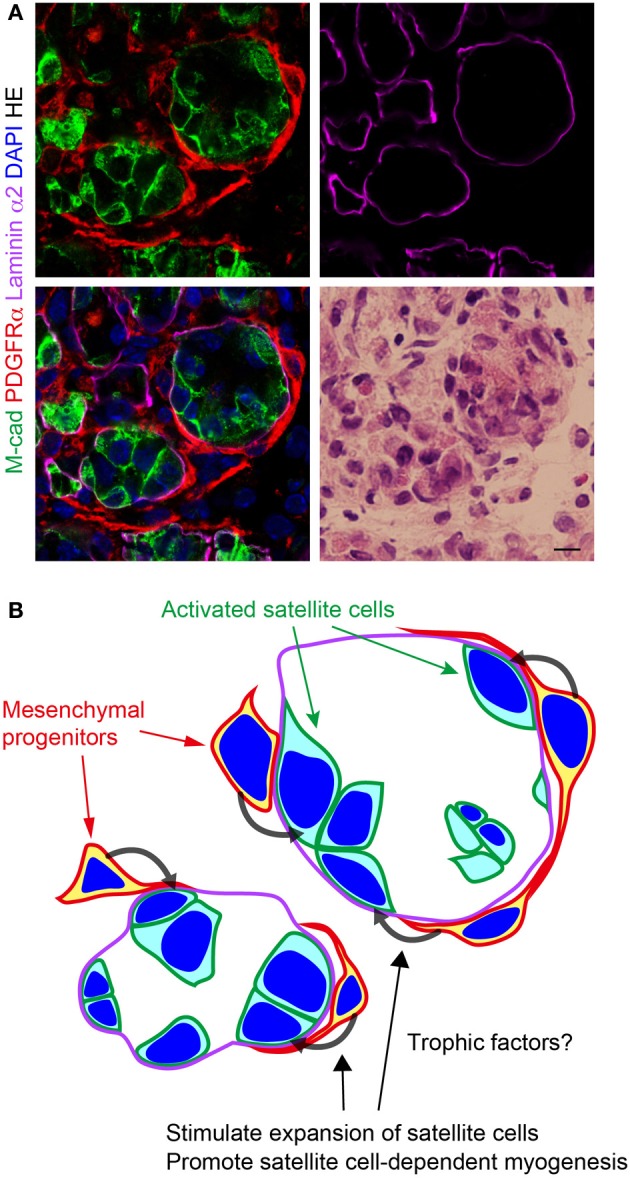
**Localization of PDGFRα^+^ mesenchymal progenitors in regenerating muscle. (A)** Fresh frozen section of regenerating muscle subjected to immunofluorescence staining for M-cadherin (M-cad), PDGFRα, and laminin α2, and subsequently to HE staining. Scale bar: 10 μm. **(B)** Schematic view of **(A)**. Mesenchymal progenitors encircle the sheath of basement membrane in which satellite cells undergo active myogenesis. Mesenchymal progenitors stimulate satellite cell expansion and promote satellite cell-dependent myogenesis. Factors responsible for these effects remain to be identified.

The pathological relevance of nonmyogenic mesenchymal progenitors to muscle diseases leads to the idea that targeting these cells can be an excellent therapeutic strategy for the treatment of muscle disorders. However, this idea should be considered carefully, because nonmyogenic mesenchymal progenitors are also present in normal healthy muscle. In fact, we found that the number of these cells significantly increased during the muscle regeneration induced by CTX injection (Uezumi et al., 2010). As described earlier, CTX injection elicits successful muscle regeneration that is not accompanied by fat infiltration and fibrosis. Thus, mesenchymal progenitors decreased in number without making fatty and fibrous connective tissue as muscle regeneration proceeded. Intriguingly, they encircled the sheath of the basement membrane in which satellite cells undergo active myogenesis during muscle regeneration (Uezumi et al., 2010) (Figure 3). These findings suggest the interaction between mesenchymal progenitors and activated satellite cells. To gain insight into the interaction between them, we employed a co-culture system and found that adipogenesis of mesenchymal progenitors was strongly inhibited by the presence of myogenic cells derived from satellite cells (Uezumi et al., 2010). Rossi and co-workers demonstrated the complementary effect of this interaction. They showed that satellite cell-dependent myogenesis is promoted by mesenchymal progenitors in the co-culture (Joe et al., 2010).

The *in vivo* significance of the interaction between two types of cells was elegantly demonstrated by Kardon and colleagues. They first found that the transcription factor Tcf4 is expressed in lateral plate-derived limb mesodermal cells distinct from myogenic cells (Kardon et al., 2003). They explored the identity of Tcf4^+^ cells and revealed that Tcf4 identifies connective tissue fibroblasts that are closely associated with skeletal muscles during development and in adulthood. By utilizing Tcf4-Cre mice, which allow genetic manipulation of connective tissue fibroblasts, fibroblasts were shown to promote slow myogenesis, the switch from fetal to adult muscle, and myoblast fusion (Mathew et al., 2011). This study clearly indicated that connective tissue fibroblasts are a critical regulator of muscle development. Roles for connective tissue fibroblasts have been further explored in adult muscle regeneration. Tcf4^+^ fibroblasts were specifically ablated using Tcf4-CreER^T2^ knock-in mice that allow conditional gene manipulation in connective tissue fibroblasts. Fibroblast-ablated mice showed impaired muscle regeneration with premature satellite cell differentiation, depletion of the early pool of satellite cells, and smaller regenerated myofibers (Murphy et al., 2011). This was the first *in vivo* demonstration of the importance of connective tissue fibroblasts as the niche regulating satellite cell expansion during regeneration. Connective tissue fibroblasts appear to have more impact in regulating muscle regeneration because the ablation efficiency of Tcf4^+^ cells was about 40% in this study. The authors also showed that specific ablation of satellite cells resulted in a complete loss of regenerated myofibers, and, importantly, misregulation of fibroblasts, leading to a dramatic increase in fatty and fibrous connective tissue (Murphy et al., 2011). Thus, reciprocal interaction between the two types of cells is critical for efficient and effective muscle regeneration. A direct relationship between mesenchymal progenitors and Tcf4^+^ fibroblasts remains to be demonstrated. However, Tcf4^+^ fibroblasts express PDGFRα (Murphy et al., 2011), a marker of mesenchymal progenitors, and accumulating evidence suggests that mesenchymal progenitors and so-called fibroblasts share much more in common than previously recognized (Sudo et al., 2007; Haniffa et al., 2009). Therefore, these cells might be largely overlapping.

Heredia et al. reported that IL-4 signaling inhibits adipogenesis and stimulates the support function of mesenchymal progenitors for successful muscle regeneration (Heredia et al., 2013). IL-4 signaling also elicited phagocytic activity in mesenchymal progenitors and promoted the clearance of necrotic fibers by mesenchymal progenitors (Heredia et al., 2013). This study provided the regulatory mechanism of mesenchymal progenitors during muscle regeneration. IL-4 is also probably best known as the canonical Th2 effector cytokine and a critical developmental determinant that promotes Th2 response but inhibits Th1 response (Swain et al., 1990). Genetic background is known to greatly affect the nature of the Th cell response. BALB/c and DBA/2 mice are well known as strains with a high Th2 bias and as high producers of IL-4, and conversely, C57BL/6 and C57BL/10 mice are well known as the strains with low Th2 bias and as low producers of IL-4 (Reiner and Locksley, 1995; Bix et al., 1998; Yagi et al., 2002; Okamoto et al., 2009). Genetic background is also known to affect muscle regeneration potential. Importantly, BALB/c and DBA/2 mice show impaired muscle regeneration with adipocyte infiltration even in the CTX model that never induces fatty degeneration in C57BL/6 or C57BL/10 mice (Fukada et al., 2010). Therefore, muscle regeneration ability appears to correlate inversely with IL-4 production ability. Although self-renewal capacity of satellite cells was diminished in DBA/2 mice (Fukada et al., 2010), it still remains a mystery why high IL-4-producing strains, BALB/c and DBA/2 mice, readily develop fatty connective tissue after CTX-induced injury. Elucidation of the detailed mechanisms by which the phenotype of mesenchymal progenitors is regulated during muscle regeneration requires further investigation.

## Roles for nonmyogenic mesenchymal progenitors in steady state homeostasis of skeletal muscle

Nonmyogenic mesenchymal progenitors may have roles in steady state homeostasis of skeletal muscle. Collagen type VI, along with the fibrillins, is one of the microfibrillar components of the ECM. Collagen VI is found in a wide variety of extracellular matrices, including muscle, skin, tendon, cartilage, intervertebral discs, lens, internal organs, and blood vessels. Collagen VI consists of three products encoded by *COL6A1*, *COL6A2*, and *COL6A3*. One of each of the three α-chain subunits encoded by *COL6A1*, *COL6A2*, and *COL6A3* combine to form the collagen VI monomer. Within the cells, these monomers associate to form dimers, which pair up into tetramers. These tetramers are then secreted into the ECM, where they assemble to form the microfibrillar structures (Bonnemann, 2011). Collagen VI microfibrils interact with collagen I, collagen IV, and with a variety of proteoglycans such as biglycan and decorin (Voermans et al., 2008). Collagen VI occurs in both the basal lamina and the reticular lamina of muscle, and therefore is an important component of endomysium and perimysium of skeletal muscle. The functional significance of collagen VI in skeletal muscle is evident as mutations in collagen VI genes cause Ullrich congenital muscular dystrophy (UCMD) and Bethlem myopathy (Bonnemann, 2011). Disease symptoms are typically more severe in UCMD than in Bethlem myopathy. UCMD patients show progressive loss of individual muscle fibers and muscle mass, and proliferation of connective and adipose tissue. The precise mechanisms leading to reduced fiber size are currently unknown. One of the unique features of collagen VI is its regulated expression. It has been demonstrated that collagen VI is largely generated by interstitial mesenchymal cells but not by myogenic cells (Zou et al., 2008). An enhancer region of the *Col6a1* gene that is required for activation of transcription in interstitial mesenchymal cells associated skeletal muscle was identified (Braghetta et al., 2008). Using reporter mice that carry this enhancer region, the expression *Col6a1* in interstitial mesenchymal cells but not in myogenic cells was confirmed. Interestingly, it has been demonstrated that the expression of Col6a1 in mesenchymal cells associated with skeletal muscle is a consequence of an inductive process whereby myogenic cells activate a specific enhancer region in mesenchymal cells by releasing a diffusible factor; in addition, only mesenchymal cells from skeletal muscle can respond to this inductive signal (Braghetta et al., 2008). Although detailed characterization of collagen VI-producing cells has not been done, their interstitial localization and mesenchymal nature suggest that these cells are closely related to mesenchymal progenitors. Thus, it is highly possible that mesenchymal progenitors play a key role in supporting muscle fibers by producing collagen VI under the inductive cue from muscle cells. Recent study extended the importance of collagen VI by demonstrating that collagen VI also works as a key component of the satellite cell niche (Urciuolo et al., 2013).

The roles for mesenchymal progenitors in the maintenance of muscle fibers have been directly demonstrated by ablating fibroblast activation protein-α (FAP)-expressing stromal cells (Roberts et al., 2013). FAP is the type II membrane dipeptidylpeptidase, and its expression has been reported to associate with fibroblasts in the tumor stroma (Garin-Chesa et al., 1990). Note that FAP differs from FAPs, fibro/adipogenic progenitors, described earlier. FAP^+^ stromal fibroblasts suppress the immune response to tumors, and elimination of FAP^+^ fibroblasts from tumor stroma unmasks the immune response to cancer and allows the immune system to attack tumors (Kraman et al., 2010). Therefore, targeting FAP^+^ stromal cells may be a promising strategy to combat cancer. However, a contraindication to any potential cancer therapy that indiscriminately depletes FAP^+^ cells might be their presence in normal tissues. To investigate the occurrence and function of FAP^+^ stromal cells in normal tissues, Roberts et al. generated transgenic mice that permit both the bioluminescent imaging of FAP^+^ cells and their conditional ablation (Roberts et al., 2013). Using this mouse line, FAP^+^ cells were found to reside in almost all tissues of the adult mouse. Surprisingly, ablation of FAP^+^ cells caused significant loss of muscle mass and hypocellularity of the bone marrow, revealing their essential functions in maintaining normal muscle mass and hematopoiesis, respectively. The loss of skeletal muscle mass was attributed to the atrophy of myofibers, and was accompanied by a persistent decrease in follistatin (Fst) and laminin α2 expression and a transient increase in atrogin-1 and MuRF1 mRNA levels. Fst is an inhibitor of myostatin (Mstn), a negative regulator of muscle mass (McPherron et al., 1997). Systemic overexpression of Mstn was reported to induce cachexia (Zimmers et al., 2002), and conversely, Fst overexpression led to dramatic increase in muscle mass (Lee and McPherron, 2001). Fst has been shown to inhibit other TGF-β family members in addition to myostatin to regulate muscle size (Lee, 2007; Lee et al., 2010). Laminin α2 is a component of muscle basal lamina and mutations in the laminin α2 gene cause congenital muscular dystrophy, in which impaired anchoring of myofibers in the ECM results in impaired membrane stability and massive muscle fiber degeneration during early infancy (Hayashi et al., 2001). Laminin α2 deficiency was also reported to induce the upregulation of key ubiquitin ligases atrogin-1 and MuRF1 (Carmignac et al., 2011). Gene expression analysis of sorted cells confirmed that FAP^+^ stromal cells are the major source of both Fst and laminin α2 in skeletal muscle (Roberts et al., 2013). Therefore, the ablation of FAP^+^ stromal cells was directly responsible for the decrease in the expression of Fst and laminin α2 in muscle, which can be considered to be the basis of the loss of muscle mass and the increase of atrogin-1 and MuRF1. Intriguingly, FAP^+^ stromal cells in muscle uniformly express PDGFRα, Sca-1 and CD90 (Roberts et al., 2013), which are shared cell surface markers with mesenchymal progenitors (Uezumi et al., 2010), and both cells are localized to the interstitial spaces of muscle tissue (Uezumi et al., 2010; Roberts et al., 2013). Thus, FAP^+^ stromal cells appear nearly identical to mesenchymal progenitors. A further important finding was that these cells undergo considerable alterations in a cachectic condition (Roberts et al., 2013). Under a cachectic condition, the FAP-dependent bioluminescence in skeletal muscle was significantly reduced, and the downregulation of Fst and laminin α2 and the upregulation of atrogin-1 and MuRF1 again accompanied dramatic loss of muscle mass. Decreased FAP-dependent bioluminescence suggests that mesenchymal progenitors decreased in number or mesenchymal progenitors lost the expression of FAP in cachectic muscle. The latter should be the case because PDGFRα^+^Sca-1^+^ cells were shown to increase in number in cachectic muscle (He et al., 2013). Collectively, mesenchymal progenitors can play vital roles in the maintenance of muscle fibers by producing trophic factors such as collagen VI, Fst, and laminin α2 in a steady physiological condition (Figure 1), and their support functions can be severely deteriorated in cancer cachexia.

## Lessons from bone marrow

Mesenchymal stem cells (MSCs) or mesenchymal progenitors were initially identified in bone marrow. Due to the ease of their isolation and their extensive proliferation and differentiation potentials, bone marrow MSCs have been expected as a source of cells for potential use in cell-based therapy and are being introduced into a clinical setting (Abdallah and Kassem, 2008). Nevertheless, the actual identity of bone marrow MSCs remains largely unknown.

Several recent studies documented *in vivo* functions of MSCs for constructing the niche of hematopoietic stem cells (HSCs). Although several cell types in bone marrow are suggested to play a role in forming the HSC niche (Frenette et al., 2013), we focus on MSCs in this article. MSCs were reported to support hematopoiesis and colocalize with HSCs throughout ontogeny (Mendes et al., 2005). In adult bone marrow, the expression of intermediate filament nestin has been shown to identify MSCs in close contact with the vasculature and HSCs (Mendez-Ferrer et al., 2010). Perivascular nestin^+^ MSCs highly express HSC maintenance genes such as *Cxcl12*, *Scf*, and *Angpt1*, and ablation of nestin^+^ MSCs rapidly reduces the concentration of HSCs in the bone marrow. A chemokine Cxcl12 plays a crucial role in maintaining HSC function and Stem cell factor (Scf), a c-kit ligand, is required to sustain hematopoiesis. Cxcl12 is produced mainly by reticular cells scattered throughout the bone marrow, and these reticular cells were termed Cxcl12-abundant reticular (CAR) cells (Sugiyama et al., 2006). Using genetically engineered mice in which a transgene encoding the diphtheria toxin receptor-GFP fusion protein is knocked into the *Cxcl12* locus, CAR cells were shown to be required for proliferation of HSCs and lymphoid and erythroid progenitors, as well as maintenance of HSCs in an undifferentiated state (Omatsu et al., 2010). CAR cells were also shown to produce most of the Scf and Cxcl12 in the bone marrow. Interestingly, CAR cells possess the potential to differentiate into adipocytes and osteoblasts (Omatsu et al., 2010), indicating that cells with MSC activity possess HSC niche activity. FAP is expressed in multipotent bone marrow stromal cells that express PDGFRα, Sca-1, Cxcl12, and Scf (Roberts et al., 2013; Tran et al., 2013). Elimination of FAP^+^ cells has been shown to induce severe bone marrow hypocellularity (Roberts et al., 2013; Tran et al., 2013). FAP^+^ cell-depleted mice showed anemia and suppressed B-lymphopoiesis and erythropoiesis although HSC frequency was not changed in these mice (Roberts et al., 2013). Leptin receptor (*Lepr*)–expressing perivascular stromal cells were shown to be one of the major sources of Scf and required to maintain HSCs (Ding et al., 2012). However, MSC activity of *Lepr*^+^ cells was not examined. The *Cxcl12* gene was knocked out conditionally using several Cre lines to clarify the importance of Cxcl12 expression in several different candidate niche cells (Greenbaum et al., 2013). As a consequence, it was demonstrated that HSCs and common lymphoid progenitors are maintained by Cxcl12 produced from Prx1-Cre-targeted cells. The paired-related homeobox gene-1 (Prx1) is expressed in the early limb bud mesenchyme and Prx1-Cre targets all mesenchymal cells in the limb bud (Logan et al., 2002). As in muscle, MSCs are enriched in PDGFRα^+^Sca-1^+^ cells in bone marrow (Morikawa et al., 2009). Importantly, only Prx1-targeted PDGFRα^+^Sca-1^+^ cells exhibited colony-forming unit-fibroblast activity and showed osteogenic and adipogenic differentiation, indicating that this subset is a highly enriched population of MSCs. Maintenance of committed B cell precursors is dependent on Cxcl12 from CAR cells and/or osteoblasts, and retention of hematopoietic progenitor cells in the bone marrow is dependent on Cxcl12 from CAR cells. These results showed the complexity of the niche microenvironment in the bone marrow and suggest that distinct stromal niche cells regulate specific hematopoietic stem/progenitor populations. Naveiras et al. reported an interesting finding. They showed that adipocyte-rich vertebrae of the mouse tail have reduced HSC frequency compared with adipocyte-free vertebrae of the thorax (Naveiras et al., 2009). Tail vertebrae of lipodystrophic mice showed normal HSC frequency, indicating that adipocytes act as negative regulators of the hematopoietic microenvironment. Because MSCs can produce adipocytes, this finding suggests the notion that their aberrant differentiation can negatively affect homeostasis of parenchymal cells. Among secretory proteins from adipocytes, adiponectin has been demonstrated to suppress the growth of myelomonocytic progenitors and the functions of macrophages (Yokota et al., 2000). Pinho et al. extended these findings to humans (Pinho et al., 2013). PDGFRα^+^CD51^+^ cells in human bone marrow represent a cell population enriched for MSCs and capable of expanding human hematopoietic stem and progenitor cells. These lines of evidence suggest that the intrinsic function of MSCs in bone marrow is to act as a niche for HSCs.

## Concluding remarks

Mesenchymal stem/progenitor cells are reported to exist in almost all organs of both mice and humans (Da Silva Meirelles et al., 2006; Crisan et al., 2008). Although *in vitro* multipotency toward adipogenic, osteogenic, and chondrogenic lineages is a hallmark of MSCs, there is no evidence that fat, bone, or cartilage are continuously generated in most organs where MSCs reside. Therefore, it seems unlikely that differentiating into a certain lineage is an intrinsic *in vivo* function of MSCs. Instead, it is tempting to speculate that these cells exert support functions for parenchymal cells of the tissue where they reside, as reviewed in this paper. Their differentiation might be undesired in most cases because adipogenic differentiation has a negative impact on hematopoiesis in bone marrow (Naveiras et al., 2009). Interestingly, pancreas-derived mesenchymal cells have a greater ability to support ES cell-derived pancreatic progenitors than the mesenchymal cells derived from other organs (Sneddon et al., 2012). Thus, mesenchymal stem/progenitor cells in certain tissues might be specialized to suitably sustain the parenchyma of that tissue. Because specific expression of collagen VI in muscle mesenchymal cells requires induction cues from myogenic cells (Braghetta et al., 2008), such a specialization might be reciprocally conducted by parenchymal cells. Exploring the detailed mechanism of parenchymal-mesenchymal interactions is an important task in a future study.

### Conflict of interest statement

The authors declare that the research was conducted in the absence of any commercial or financial relationships that could be construed as a potential conflict of interest.
